# Relationship between the complement system and serum lipid profile in patients with rheumatoid arthritis

**DOI:** 10.3389/fimmu.2024.1420292

**Published:** 2024-07-12

**Authors:** Dara Rodríguez-González, María García-González, Fuensanta Gómez-Bernal, Juan C. Quevedo-Abeledo, Agustín F. González-Rivero, Alejandro Jiménez-Sosa, Elena González-López, Elena Heras-Recuero, J. Gonzalo Ocejo-Vinyals, Miguel Á. González-Gay, Iván Ferraz-Amaro

**Affiliations:** ^1^ Division of Central Laboratory, Hospital Universitario de Canarias, Santa Cruz de Tenerife, Spain; ^2^ Division of Rheumatology , Hospital Universitario de Canarias, Santa Cruz de Tenerife, Spain; ^3^ Division of Rheumatology, Hospital Doctor Negrín, Las Palmas de Gran Canaria, Spain; ^4^ Research Unit , Hospital Universitario de Canarias, Santa Cruz de Tenerife, Spain; ^5^ Division of Immunology, Hospital Universitario Marqués de Valdecilla, Instituto de Investigación sanitaria Marqués de Valdecilla (IDIVAL), Santander, Spain; ^6^ Division of Rheumatology, Instituto de Investigación Sanitaria (IIS)-Fundación Jiménez Díaz, Madrid, Spain; ^7^ Department of Medicine and Psychiatry, University of Cantabria, Santander, Spain; ^8^ Department of Internal Medicine, University of La Laguna (ULL), Santa Cruz de Tenerife, Spain

**Keywords:** rheumatoid arthritis, complement system, lipids, dyslipidemia, inflammation

## Abstract

**Background:**

The complement system has been linked to the etiopathogenesis of rheumatoid arthritis (RA). Patients with RA exhibit a dysregulated profile of lipid molecules, which has been attributed to the inflammation present in the disease. In this study, we aimed to evaluate the association between a comprehensive assessment of the complement system and the lipid profile of patients with RA.

**Methods:**

430 patients with RA were recruited. New-generation techniques were employed to conduct functional assays of the three pathways of the complement system. Serum levels of various complement components such as C1q, factor D, properdin, lectin, C1-inhibitor, C2, C4, C4b, C3, C3a, C5, C5a, and C9 were assessed. Furthermore, a complete pattern of lipid molecules was measured including high (HDL), low-density lipoproteins (LDL), and lipoprotein (a). Multivariable linear regression analysis was conducted to investigate the association between the complement system and lipid profile in RA patients.

**Results:**

After multivariable analysis, several noteworthy associations emerged between the complement system and lipid molecules. Notably, complement components most strongly linked to the lipid profile were C1q and properdin, representing the upstream classical and alternative pathways, along with C3 from the common cascade. These associations demonstrated significance and positivity concerning total cholesterol, LDL, atherogenic index, apolipoprotein B, and lipoprotein(a), suggesting a connection with an unfavorable lipid profile. Interestingly, complement functional assays of the three pathways and activated products such as C3a and C5a showed no correlation with the lipid pattern.

**Conclusion:**

The correlation between the complement system and lipid molecule patterns is pronounced in patients with RA. This relationship is predominantly positive and primarily associated with upstream complement components rather than activated ones.

## Introduction

The complement (C) system is integral to the innate immune response, working in conjunction with antibody-mediated processes. It is an omnipresent and essential component of the immune system, playing a critical role in various biological contexts. It comprises a network of proteins that work synergistically to defend against pathogens by promoting inflammation, opsonization, and cell lysis. Beyond its traditional role in immune defense, the C system is involved in numerous physiological processes, including tissue regeneration, clearance of immune complexes, and maintenance of homeostasis. Its relevance extends to various pathological conditions such as autoimmune diseases, where dysregulation can contribute to disease progression. The C system’s broad functional spectrum underscores its importance in maintaining health and responding to disease. This system is structured into three distinct but interconnected activation pathways: the classical, alternative, and lectin cascades. Moreover, there is a common terminal lytic pathway and a sophisticated network of regulators and receptors (1). Each pathway is activated through distinct mechanisms, yet all converge to activate C3 and its subsequent deposition as C3b, which is a central event in C activation. The classical pathway is initiated by antibodies, while the lectin pathway is specialized in rapidly recognizing repetitive carbohydrate patterns on the surfaces of microbial pathogens. In contrast, the alternative pathway serves as an ancient surveillance system and represents the original extracellular complement route. The alternative pathway can be activated without the need for antibodies or lectins and operates continuously at a low level due to the presence of a labile thioester bond at C3, a phenomenon known as “tick-over”.

Rheumatoid arthritis (RA) is a symmetric, inflammatory, peripheral polyarthritis of unknown etiology. It typically leads to joint destruction through the erosion of cartilage and bone.

Moreover, RA patients exhibit a higher prevalence of atherosclerosis compared to healthy controls (2), resulting in an elevated incidence of cardiovascular events such as stroke, myocardial infarctions, and cardiac deaths when compared to the general population (3, 4). It is postulated that the presence of chronic inflammation in RA may enhance the development of atherosclerosis. In contrast, suppression of inflammation may have a beneficial effect on preventing the progression of subclinical atherosclerosis (5). Additionally, RA affects lipid profiles. Consequently, alterations in serum lipids among RA patients are commonly observed in clinical evaluations, arising from the systemic inflammatory state and pharmacological treatments for RA (6). Generally, these lipid molecule changes involve a reduction in total cholesterol, low-density lipoprotein cholesterol (LDL), and high-density lipoprotein cholesterol (HDL) levels in untreated patients before and during active periods (7). Similarly, the serum lipoprotein (a) concentration in RA patients significantly increases, and the apolipoprotein B (Apo B)/apolipoprotein A-1 (Apo A-1) ratio is also notably higher compared to the control group (6). The exact mechanisms linking the pathophysiology and inflammation of patients with RA to these modifications in lipid pattern are not fully understood to date.

The evidence for C activation as a mediator of joint and extra-articular inflammation in RA is strong (8). For example, elevated serum levels of C1q, attributed to genetic variations, may increase susceptibility to developing RA, potentially due to a greater capacity for activation through the classical pathway (9). Furthermore, the involvement of local intra-articular C activation in RA is evidenced by the observation of decreased total hemolytic C activity and decreased levels of specific components in synovial fluid (8). C5a and C3a have been described to play crucial roles in the pathogenesis of RA. C5a is involved in the effector phase of synovial infiltration and joint destruction, making it a potential therapeutic target (10), and it is also a key mediator of neutrophil accumulation and acute joint swelling and pain (11). Likewise, C5a contributes to the production of pro-inflammatory factors and the development of undesirable new blood vessels, leading to bone and joint destruction (12). The critical role of C5a in initiating neutrophil-mediated autoimmune inflammation in the joint and skin further underscores its significance in the pathogenesis of rheumatoid arthritis (13). Furthermore, unpublished observations from our group indicate that disease activity in RA leads to an increase in C molecules and functional C assays but, conversely, positivity for rheumatoid factor or anti- citrullinated antibodies was associated with C consumption.

The association between serum lipid profiles and inflammation-related markers in RA is an issue of considerable interest. In RA, chronic inflammation not only affects joints but also disrupts lipid metabolism, leading to abnormal lipid and lipoprotein profiles. The C system, integral to immune response regulation, is often overactive in RA and can influence lipid metabolism. Understanding this correlation is crucial as it would shed light on how inflammation impacts lipid levels and contributes to cardiovascular risk in RA patients. This insight could guide new therapeutic approaches targeting both inflammation and lipid abnormalities. For our study, we employed advanced next-generation functional assays to evaluate the three pathways of the C system. Additionally, we analyzed various components of the C system associated with all three cascades, including enzymatically produced fragments and serum regulators. Our primary objective was to elucidate the relationships between the functional levels of the three C cascades and specific elements within these pathways. Additionally, we conducted a thorough assessment of the lipid profile, encompassing total, LDL, and HDL cholesterols, as well as lipoprotein (a).

## Materials and methods

### Study participants

Cross-sectional study that included 430 patients with RA recruited consecutively. All participants were 18 years old or older and met the 2010 ACR/EULAR classification criteria (14). They had been diagnosed by rheumatologists and were undergoing regular follow-up appointments at rheumatology outpatient clinics. To be included in the present study, participants were required to have a duration of RA disease of at least one year. As glucocorticoids are frequently utilized in RA treatment, patients receiving prednisone or an equivalent dose of ≤10 mg/day were eligible for participation in the study. Patients with a history of cancer or any other chronic diseases such as hypothyroidism, heart or respiratory diseases, nephrotic syndrome, as well as those displaying evidence of active infection, were excluded from the study. The study protocol was approved by the Institutional Review Committee at Hospital Universitario de Canarias and at Hospital Universitario Doctor Negrín (both in Spain), and all subjects provided informed written consent (approval no. 2019–452-1). All research activities were carried out in strict compliance with applicable guidelines and regulations, and in accordance with the principles set forth in the Declaration of Helsinki.

### Data collection, laboratory assessments and carotid ultrasound evaluation

Participants enrolled in the study underwent a comprehensive examination, which included completing a questionnaire regarding cardiovascular risk factors and medication usage. A thorough physical examination was conducted, which involved measurements such as body-mass index (BMI) calculated as weight in kilograms divided by the square of the height in meters, abdominal circumference, and assessment of systolic and diastolic blood pressure under standardized conditions. Additionally, information regarding smoking, diabetes, and hypertension was gathered. Specific diagnoses and medication details were verified through a review of medical records.

Blood samples were collected from fasting patients (9–12 hours) via venipuncture into vacutainer tubes. For serum collection, blood samples were allowed to clot at room temperature for 30 minutes, followed by centrifugation to separate the serum. The serum samples were then transferred to labeled secondary tubes and stored at 2–8°C for short-term storage or frozen at -80°C for long-term storage. All samples were transported to the laboratory on ice to maintain stability. Proper labeling and documentation accompanied each sample to ensure accurate identification and analysis. Cholesterol, triglycerides, and HDL cholesterol were measured using the enzymatic colorimetric assay (Roche) in serum. Lipoproteins were assessed using a quantitative immunoturbidimetric assay (Roche) in serum. Cholesterol ranged from 0.08 to 20.7 mmol/l (intra-assay coefficient of variation of 0.3%); triglycerides ranged from 4 to 1.000 mg/dl (intra-assay coefficient of variation of 1.8%); and HDL cholesterol ranged from 3 to 120 mg/dl (intra-assay coefficient of variation of 0.9%). The atherogenic index was calculated using the total cholesterol: HDL cholesterol ratio according to the Castelli formula. LDL cholesterol was calculated using the Friedewald formula. Dyslipidemia was defined if one of the following was present: total cholesterol > 200 mg/dL, triglycerides > 150 mg/dL, HDL cholesterol < 40 in men or <50 mg/dL in women, or LDL cholesterol > 130 mg/dL. A standard technique was used to measure the erythrocyte sedimentation rate (ESR) and high-sensitivity C-reactive protein (CRP). Disease activity in patients with RA was measured using the Disease Activity Score (DAS28) in 28 joints (15), the Clinical Disease Activity Index (CDAI) (16) and the Simple Disease Activity Index (SDAI) (17). DAS28-ESR and DAS28-CRP were classified into distinct categories based on predefined thresholds: remission (<2.6), low (>2.6 to 3.2), moderate (>3.2 to 5.1), or high disease activity (>5.1) as previously described (18). Likewise, SDAI categories were defined as follows: remission (<3.3), moderate disease activity (<11), high disease activity (<26), and very high disease activity (>26). Concurrently, the CDAI was categorized into remission (<2.8), moderate disease activity (<10), high disease activity (<22), and very high disease activity (>22). These categorizations adhere to established criteria (19).

### Complement assessments

The SVAR functional C assays under the Wieslab^®^ brand (Sweden) were used to assess classical, alternative and lectin pathways activity. These tests combine principles from the hemolytic assay for C function with the use of labeled antibodies that specifically target the neoantigen produced as a result of C activation. The quantity of neoantigen generated is directly proportional to the functional activity of the C pathways. Microtiter strip wells are coated with classical, alternative or lectin pathway-specific activators. In this process, the patient’s serum is diluted with a specific blocker to ensure activation of only the studied C pathway. During the incubation of the diluted patient serum in the wells, the specific coating activates C. Subsequently, the wells are washed, and the presence of C5b-9 is detected using an alkaline phosphatase-labeled specific antibody against the neoantigen expressed during membrane attack complex (MAC) formation. Following an additional washing step, specific antibodies are detected by incubating with an alkaline phosphatase substrate solution. The intensity of the color developed correlates with the amount of C activation and is measured in terms of absorbance (optical density). The quantity of formed Membrane Attack Complex (MAC) neo-epitope reflects the activity of the C cascade. The result is expressed semi-quantitatively by calculating the optical density ratio between a positive control and the sample. It is crucial to note that for the classical, alternative, and lectin cascade values, lower levels indicate a higher activation of the respective pathway. Wieslab^®^ has validated these functional assays by studying their correlation and concordance with the classical CH50 and AH50 hemolytic tests (https://www.svarlifescience.com/). Additionally, C individual elements were assessed through MILLIPLEX^®^ map Multiplex Detection (MERCK^®^, Cat. No. HCMP1MAG-19K and No. HCMP2MAG-19K). To achieve a comprehensive characterization of all C pathways, panels were devised to evaluate various components, including C1q (classical pathway), factor D and properdin (alternative pathway), lectin (lectin pathway), C1 inhibitor, C2, C4, and C4b (classical and lectin pathways), C3, C3a, and C4b (common pathway), as well as C5, C5a, and C9 (terminal pathway). Both intra- and inter-coefficients of variability for these assays were maintained below 10%.

### Statistical analysis

The required sample size for a one-sample correlation test was calculated using Fisher’s z test method. A significance level (alpha) of 0.05 and a statistical power of 0.80 were set for the analysis. Since there are not previous report that assessed the correlations between C particles to lipid molecules, the expected correlation under the null hypothesis (H0) was set at r0 = 0.15, while the alternative hypothesis (Ha) assumed a correlation of ra = 0.30. Based on these parameters, the estimated sample size was N = 316 participants. Demographic and clinical characteristics in patients with RA were described using means (standard deviation) or percentages for categorical variables. For non-normally distributed continuous variables, data were expressed as median and interquartile range (IQR). The association between lipid profile and circulating C system molecules and pathways was initially analyzed using Spearman’s rho correlation coefficients. Subsequently, multivariable linear regression analysis was conducted to examine the relationship between C system routes and elements (independent variables) and lipid profile (dependent variable), while adjusting for covariates. For the construction of a heatmap of multivariable associations, standardized beta coefficients were calculated and plotted. Using standardized beta coefficients, rather than non-standardized ones, facilitates comparison between beta coefficients in multiple associations. All analyses were conducted with a 5% two-sided significance level using Stata software, version 17/BE (StataCorp, College Station, TX, USA). P-values <0.05 were considered statistically significant.

## Results

### Demographic and disease-related data

This study included a total of 430 patients diagnosed with RA. Demographic- and disease-related characteristics of the participants are shown in **Supplementary Table 1**. The study population had a mean age of 55 ± 10 years, with 81% of the participants being women. The median duration of the disease was 8 years (interquartile range, IQR, 4–15). At the time of the study, the mean values of CRP and ESR were 2.7 mg/l (IQR 1.3–6.1) and 18 mm/1^st^ hour (IQR 7–32), respectively. Rheumatoid factor was positive in 72% of patients, and 65% were positive for anti-citrullinated protein autoantibodies (ACPA). The disease activity, as measured by DAS28-ESR, was 3.1 ± 1.4. According to this score, 40% of the patients met the criteria for remission, while 18% and 42% were categorized in the low and moderate/high disease activity groups, respectively. The DAS28-CRP had a value of 2.7 ± 1.1, and SDAI and CDAI were 12 (IQR 7–19) and 8 (IQR 4–14), respectively. Thirty-six percent of the patients were undergoing treatment with prednisone, while 87% were receiving at least one conventional disease-modifying antirheumatic drug (DMARD) of any type, with methotrexate being the most commonly prescribed (73%). Nineteen percent of the patients were receiving anti-tumor necrosis factor therapies. The frequency of usage of other treatments and historical disease-related data can be found in **Supplementary Table 1**.

Functional C assays of the classical, alternative and lectin pathways, and single C components, C1q, C1-inhibitor, C2, C4, C4b, C3, C3a, C5, C5a, and C9, and factor D and I, properdin and lectin serum values are presented in **Supplementary Table 2**. Lipid profile molecules values are also provided in **Supplementary Table 2**.

### Univariable and multivariable analysis of the relationship between complement system and lipid profile

**Figure 1:**
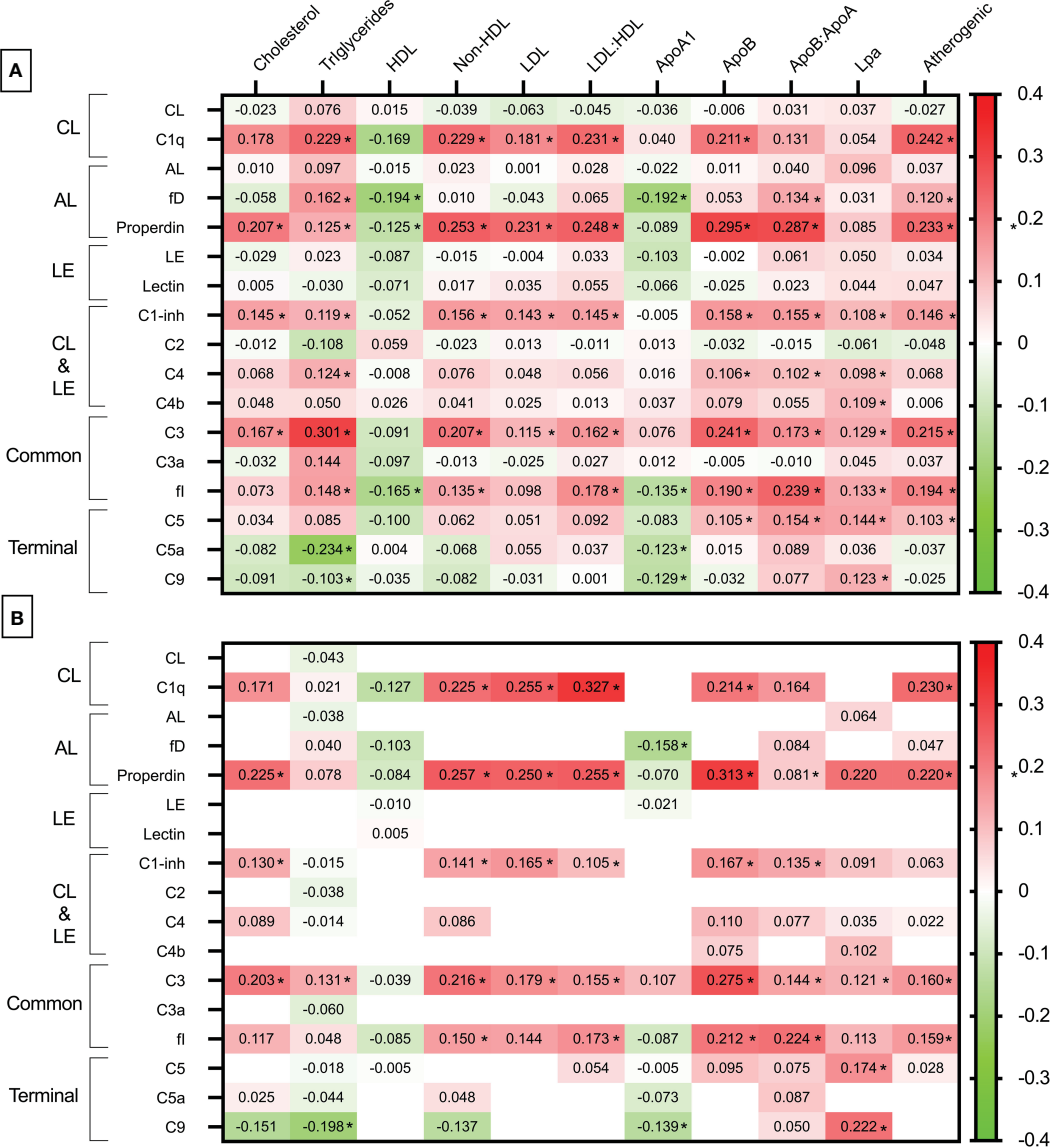
Heatmap of the relationship between complement system and lipid profile. **(A)** Spearman’srho correlation heatmap analysis of complement system pathways and individual particles to lipid profile molecules. **(B)** Multivariable standardized beta coefficients heatmap of the association between complement system (independent variable) and lipid profile molecules (dependent variable) adjusted for age, sex, abdominal circumference, use of statins, anti-TNF therapies and tocilizumab, and DAS28-CRP. Only Spearman’srho correlation coefficients with a p value inferior to 0.20 in [A] are tested in the multivariable regression analysis [B]. CL, classical; LE, lectin; AL, alternative; fI, factor I; fD, factor D. Significant correlation and standardized beta coefficients with a p<0.05 are depicted as *.

Spearman’s rho correlation heatmap analysis of C system pathways and individual particles to lipid profile molecules are shown in **Figure 1A**. As observed in this correlation heatmap, most of the associations between the C system and the lipid profile were positive (in red). Only HDL and ApoA1, which are molecules with beneficial lipid properties, showed a negative relationship (in green). Many of these relationships were statistically significant. Remarkably, the functional C tests corresponding to the three pathways (CL, AL, and LE in the figure) did not show statistical significance with the lipid profile. Additionally, significant associations were predominant in the upper part of the heatmap, corresponding to the upstream C zymogens. Thus, C1q, which belongs to the early classical pathway, showed a significant relationship with all C molecules except HDL, apolipoprotein A1, and lipoprotein (a). Properdin also showed associations with all lipid profile molecules apart from apolipoprotein A1and lipoprotein (a). Additionally, C1-inhibitor, factor I, and C3a were among the particles that showed higher associations with different lipid molecules. Remarkably, the C activation products C3a and C5a did not show association with the lipid profile (**Figure 1A**). Complete Spearman’s rho correlations between C system and lipid pattern are shown in **Supplementary Table 3**.

Due to potential influences from disease activity, medications used in treatment, and various other factors, we conducted an additional multivariable linear regression analysis. In this analysis, only correlations with a p-value less than 0.20 from **Figure 1A** were subsequently adjusted and expressed as standardized beta coefficients, considering the C system as the independent variable and the lipid profile as the dependent variable. Standardized beta coefficients provide a measure of the strength and direction of the relationship between variables when they are expressed in different units or scales. Standardizing the coefficients allows for comparisons by bringing all variables to a common scale. Standardized beta coefficients in **Figure 1B** are adjusted for age, sex, abdominal circumference, use of statins, anti-TNF therapies and tocilizumab, and DAS28-CRP.

Following adjustment for covariates, numerous associations identified in the univariable analysis remained significant. Specifically, C1q retained significant and positive associations with non-HDL and LDL-cholesterol, LDL: HDL and ApoB: ApoA1 ratios, ApoB, and the atherogenic index. Also, properdin serum levels were significantly associated with total cholesterol, non-HDL, LDL and LDL: HDL ratio and atherogenic index. Several other associations identified in the univariable analysis concerning C1-inhibitor, C3, and factor I were also preserved. Again, functional C assays and activated C forms such as C3a and C5a showed no correlation with the lipid pattern (**Figure 1B**). A complete standardized beta coefficients representation between C system and lipid pattern is shown in **Supplementary Table 4**.

### Multivariable relationship between complement system and LDL>130 mg/dl, atherogenic index >4, and the presence of dyslipidemia

To ascertain how our findings correlate with specific lipid values known to impact cardiovascular disease or cardiovascular events, we examined the association of the C system with LDL>130 mg/dl, an atherogenic index >4, and the presence of dyslipidemia (defined as the presence of one of the following: total cholesterol > 200 mg/dL, triglycerides > 150 mg/dL, HDL cholesterol < 40 in men or <50 mg/dL in women, or LDL cholesterol > 130 mg/dL). This analysis was adjusted for the aforementioned potential confounders. The relationship of the C system with these outcomes remained consistent across them. Specifically, patients with LDL>130 mg/dl, an atherogenic index >4, and dyslipidemia exhibited significantly higher levels of properdin, C1-inhibitor, and C3 after adjustment. Furthermore, patients with LDL>130 mg/dl and dyslipidemia showed elevated levels of C1q, while an atherogenic index >4 was associated with increased levels of factor I (**Table 1**).

**Table 1:** Multivariable analysis of the relation of complement system routes and elements to the presence of LDL>130 mg/dl, dyslipidemia and atherogenic index.

	LDL>130 mg/dl		Dyslipidemia		Atherogenic index > 4	
	No= 262	Yes=159	No=98	Yes=324	No=268	Yes=154
Functional complement assays, %						
Classical pathway	0.7 (-4–6)	0.77	0.2 (-5–6)	0.94	-4 (-9–1)	0.14
Alternative pathway	2 (-3–7)	0.47	3 (-3–8)	0.37	-1 (-6–4)	0.66
Lectin pathway	-0.6 (-11–10)	0.91	-4 (-16–8)	0.55	-0.2 (-11–11)	0.97
Individual complement components						
Classical pathway						
C1q, mg/dl	**4 (1–7)**	**0.003**	3 (-0.8–6)	0.13	**4 (1–7)**	**0.004**
Alternative pathway						
Factor D, mg/dl	-0.004 (-0.2–0.009)	0.54	-0.003 (-0.02–0.01)	0.71	0.003 (-0.01–0.02)	0.66
Properdin, mg/dl	**0.1 (0.04–0.2)**	**0.002**	**0.1 (-0.06–0.2)**	**0.001**	**0.2 (0.08–0.2)**	**<0.001**
Lectin pathway						
Lectin, mg/dl	0.02 (-0.08–0.05)	0.15	0.008 (-0.03–0.04)	0.63	0.02 (-0.01–0.05)	0.23
Classical and lectin pathways						
C1-inhibitor, mg/dl	**2 (0.5–3)**	**0.006**	**2 (0.08–3)**	**0.039**	**2 (0.4–3)**	**0.011**
C2, mg/dl	0.7 (-1–3)	0.50	-1 (-4–1)	0.31	-0.5 (-3–2)	0.62
C4, mg/dl	0.9 (-1–3)	0.37	2 (-0.5–4)	0.14	0.9 (-1–3)	0.36
C4b, mg/dl	0.1 (-0.6–0.8)	0.75	0.5 (-0.3–1)	0.23	-0.1 (-0.8–0.5)	0.71
Common pathway						
C3, mg/dl	**8 (3–13)**	**0.002**	**7 (2–13)**	**0.013**	**8 (3–13)**	**0.002**
C3a, mg/dl	1 (-2–4)	0.48	2 (-2–5)	0.31	0.4 (-3–3)	0.77
Factor I, mg/dl	0.2 (-0.01–04)	0.063	0.2 (-0.08–0.4)	0.18	**0.3 (-0.02–0.5)**	**0.027**
Terminal pathway						
C5, mg/dl	0.04 (-0.3–0.4)	0.84	0.07 (-0.4–0.5)	0.74	0.1 (-0.2–0.5)	0.50
C5a, mg/dl	0.1 (-0.09–0.3)	0.29	0.04 (-0.2–0.3)	0.72	0.1 (-0.09–0.3)	0.27
C9, mg/dl	-0.06 (-0.2–0.04)	0.25	-0.03 (-0.2–0.09)	0.60	-0.04 (-0.1–0.07)	0.51

In this analysis, the dependent variables consist of the complement system elements, while LDL>130 mg/dl, dyslipidemia, and atherogenic indices serve as the independent variables. Non-standardized beta coefficients are adjusted for age, sex, abdominal circumference, use of statins, anti-TNF therapies and tocilizumab, and DAS28-CRP. LDL, Low-density lipoprotein. Statistically significant values are depicted in bold.

## Discussion

Understanding the mechanisms underlying inflammatory dyslipidemia in RA is crucial, given its association with an abnormal blood lipid profile. Our study represents the first exploration in the literature of the correlation between the C system and the lipid profile in RA patients. Our findings reveal an independent link between the C system and specific lipid parameters in RA. The segments of the C system implicated in this association are those upstream of the cascade and regulators, rather than its activated elements. Furthermore, the relationship between the C system and the lipid profile is positive, indicating a correlation with an unfavorable lipid pattern.

The study of the C system in RA has not received as much attention as in other diseases such as systemic lupus erythematosus, antiphospholipid syndrome, or vasculitis. Furthermore, contrary to what happens in these diseases, it seems that C components are elevated in RA rather than decreased (20, 21). This upregulation of the C system in RA may be attributed to increased hepatic production associated with inflammation, which ultimately does not result in increased C deposition or activity.

While the connection between the C system and serum lipoproteins has been investigated in the general population, such studies are lacking in RA patients. For instance, in the Diabetes and Atherosclerosis Maastricht (CODAM) cohort, associations of C3 and other components of the alternative C pathway with plasma lipoprotein subclass profile were examined (22). It was discovered that higher C3 concentrations were linked to increased levels of circulating VLDL, intermediate-density lipoproteins (IDLs) and LDL, as well as small HDL particles, but reduced levels of very large and large HDL particles. Moreover, similar albeit weaker associations were observed for properdin, factor H, factor D, and mannan-binding lectin-associated serine proteases 3 (MASP-3), while no associations were found for C3a and Bb. These observed associations were largely independent of obesity, insulin resistance, and low-grade inflammation.

In RA, our findings suggest that this relationship is independent of disease activity and is not influenced by active C products but rather by serum levels of inactive zymogens. Similarly, in a study involving a cohort of 756 unselected adults, a notable odds ratio for the likelihood of coronary heart disease was identified based on C3 serum levels (23). This relationship was proved to be independent of CRP and other standard cardiovascular risk factors. Remarkably, C3 was independently associated with serum triacylglycerols and total cholesterol. The authors suggested C3 might be actively involved in coronary atherothrombosis (23). Other studies have linked C3 to triacylglycerols (24) and C3 and C4 to triglycerides (25). However, none of these studies has conducted such a comprehensive analysis of the C system as ours, which included functional tests and elements from all three pathways. Remarkably, in our study blood lipid profile associations were found fundamentally with C1q, C1-inhibitor, properdin, C3 and factor I. Properdin is released from granules upon neutrophil activation, binds activated C3 to form a C3 convertase, and it serves a platform for efficient C3 activation by the alternative pathway (26). C1q is a component of the C system that plays a crucial role in the classical pathway of C activation. It acts as the recognition molecule for immune complexes, microbial surfaces, and apoptotic cells, initiating the activation cascade by binding to IgG or IgM antibodies that are bound to their target antigens (27). C1-inhibitor and factor I are enzymes that plays a significant role in the negative regulation of the C system. Factor I primary function is to degrade activated C components, especially C3b and C4b, into their inactive forms, C3bi and C4c respectively (28). This helps prevent excessive activation of the C system and self-injury to normal cells and tissues. Remarkably, in our study functional tests of the three cascades, as well as activated products like C3a and C5a, were not associated with lipid molecules. Additionally, C9 did not reveal any association with the blood lipid profile. Based on these findings, the pattern of association between the C system and lipid molecules in RA appears to align with the elements from the upper cascade, predominantly involving the classical and alternative pathways, rather than the lectin route. Furthermore, it seems to be represented by inactive forms of the C system rather than activated products. The relationship found for C1-inhibitor and factor I is likely due to the overexpression of these elements as a consequence of an upregulation of the entire C system.

Previous studies established a connection between lipid metabolism and the C system. For instance, evidence has been presented regarding the ability of C3-derived products to modulate the migratory and repair function of vascular smooth muscle cells, which is impaired by LDL (29). Moreover, factor H has been described to protect arteries from inflammation, ultimately preventing the formation of lipid-rich deposits (30). Treatment of human macrophages with anaphylatoxin C3a results in stimulation of C3 transcription and secretion as well as increased oxidized LDL accumulation and augmented oxidized LDL-mediated up-regulation of the C3 gene (31). Similarly, altered lipid profiles have been observed in mouse models deficient in certain C components (32).

Total cholesterol, LDL, and HDL levels in untreated patients with RA with active disease are decreased (33). In these patients the serum lipoprotein (a) concentration is increased significantly, and the ApoB: ApoA1 ratio is significantly higher than that in healthy individuals (34). This has been named ‘lipid paradox’ (7). These changes in the lipid molecules are thought to be consequence of the inflammation present in the disease (6). In our study we observed a positive, but not negative, relationship between C system and the lipid profile. However, according to our results, the lipid reduction found in RA patients who have active disease is not mediated by the C system.

We acknowledge several limitations in our study. Firstly, its cross-sectional nature limits our ability to establish causality. Additionally, the absence of control subjects in our study may be seen as a limitation. However, our primary aim was not to compare with controls as this has been addressed in previous research. Another potential limitation is that we only measured values of the lipid profile commonly used in clinical practice, without considering other parameters such as oxidized forms or precursor particles of the lipid profile. Nonetheless, our objective was to explore the relationship between the C system and lipid parameters commonly used in clinical practice, which are known to have predictive capacity for cardiovascular disease. Furthermore, a potential strength of our study was the comprehensive assessment of a wide range of lipid molecules and the detailed phenotyping of study participants, allowing for thorough adjustment for potential confounders. Lastly, we did not assess rheumatoid factor or ACPA isotypes. Therefore, multivariable analysis could not be adjusted for these isotypes.

In conclusion, patients with RA exhibit an independent relationship between the C system and a deleterious lipid profile.

## Data availability statement

The raw data supporting the conclusions of this article will be made available by the authors, without undue reservation.

## Ethics statement

The studies involving humans were approved by Comité de Ética de la Investigación del Hopsital Universitario de Canarias. The studies were conducted in accordance with the local legislation and institutional requirements. The participants provided their written informed consent to participate in this study.

## Author contributions

DR-G: Data curation, Formal analysis, Writing – review & editing. MG-G: Conceptualization, Data curation, Formal analysis, Writing – review & editing. FG-B: Data curation, Formal analysis, Writing – review & editing. JQ-A: Data curation, Writing – review & editing. AG-R: Data curation, Formal Analysis, Writing – review & editing, Project administration. AJ-S: Formal analysis, Methodology, Writing – review & editing. EG-L: Formal analysis, Writing – review & editing. EH-R: Formal analysis, Writing – review & editing. JGO-V: Formal analysis, Writing – review & editing. MG-G: Writing – original draft, Writing – review & editing. IF-A: Conceptualization, Funding acquisition, Methodology, Resources, Supervision, Writing – original draft, Writing – review & editing.
