# Supplementary material for: Relationship between the complement system and serum lipid profile in patients with rheumatoid arthritis
Source: Front Immunol. 2024 Jul 12;15:1420292. doi: 10.3389/fimmu.2024.1420292 (PMC11272461; doi:10.3389/fimmu.2024.1420292)
Supplement: Supplementary file 1 [file Table_1.docx]

| **Supplementary Table 1. Demographics and disease related data in RA patients.** | | | | | | | | |  |  |  |
| --- | --- | --- | --- | --- | --- | --- | --- | --- | --- | --- | --- |
|  |  |  | Rheumatoid arthritis | | | | |  |  |  |  |
|  |  |  | | (n=430) | |  |  |  |  |  |  |
| Age, years | | | | | 55 ± 10 | |  |  |  |  |  |
| Female, n (%) | | | | | 350 (81) | |  |  |  |  |  |
| BMI, kg/m^2^ | | | | | 28 ± 5 | |  |  |  |  |  |
| Abdominal circumference, cm | | | | | 97 ± 13 | |  |  |  |  |  |
| Hip circumference, cm | | | | | 106 ± 11 | |  |  |  |  |  |
| Abdominal to hip ratio | | | | | 0.92 ± 0.08 | |  |  |  |  |  |
| Cardiovascular risk factors, n (%) | | | | |  | |  |  |  |  |  |
|  | Current smoker | | | 93 (22) | |  |  |  |  |  |  |
|  | Obesity | | | 137 (32) | |  |  |  |  |  |  |
|  | Hypertension | | | 148 (34) | |  |  |  |  |  |  |
|  | Diabetes Mellitus | | | 54 (13) | |  |  |  |  |  |  |
| Statins | | | | | 139 (32) | |  |  |  |  |  |
| Disease related data | | | | |  | |  |  |  |  |  |
| Disease duration, years | | | | | 8 (4-15) | |  |  |  |  |  |
| CRP at time of study, mg/l | | | | | 2.7 (1.3-6.1) | |  |  |  |  |  |
| ESR at time of study, mm/1^st^ hour | | | | | 18 (7-32) | |  |  |  |  |  |
| Rheumatoid factor, n (%) | | | | | 303 (72) | |  |  |  |  |  |
| ACPA, n (%) | | | | | 253 (65) | |  |  |  |  |  |
| DAS28-ESR | | | | | 3.13 ± 1.35 | |  |  |  |  |  |
|  | Remission, n (%) | | | 166 (40) | |  |  |  |  |  |  |
|  | Low activity, n (%) | | | 76 (18) | |  |  |  |  |  |  |
|  | Moderate activity, n (%) | | | 138 (33) | |  |  |  |  |  |  |
|  | High activity, n (%) | | | 39 (9) | |  |  |  |  |  |  |
| DAS28-PCR | | | | | 2.73 ± 1.08 | |  |  |  |  |  |
|  | Remission, n (%) | | | 224 (53) | |  |  |  |  |  |  |
|  | Low activity, n (%) | | | 71 (17) | |  |  |  |  |  |  |
|  | Moderate activity, n (%) | | | 111 (26) | |  |  |  |  |  |  |
|  | High activity, n (%) | | | 15 (4) | |  |  |  |  |  |  |
| SDAI | | | | | 12 (7-19) | |  |  |  |  |  |
|  | Remission, n (%) | | | 33 (8) | |  |  |  |  |  |  |
|  | Low activity, n (%) | | | 155 (37) | |  |  |  |  |  |  |
|  | Moderate activity, n (%) | | | 179 (43) | |  |  |  |  |  |  |
|  | High activity, n (%) | | | 52 (12) | |  |  |  |  |  |  |
| CDAI | | | | | 8 (4-14) | |  |  |  |  |  |
|  | Remission, n (%) | | | 79 (19) | |  |  |  |  |  |  |
|  | Low activity, n (%) | | | 198 (47) | |  |  |  |  |  |  |
|  | Moderate activity, n (%) | | | 116 (27) | |  |  |  |  |  |  |
|  | High activity, n (%) | | | 30 (7) | |  |  |  |  |  |  |
| History of extraarticular manifestations, n (%) | | | | | 38 (10) | |  |  |  |  |  |
| Erosions, n (%) | | | | | 166 (43) | |  |  |  |  |  |
| Current drugs, n (%) | | | | |  | |  |  |  |  |  |
|  | Prednisone | | | 156 (36) | |  |  |  |  |  |  |
|  | Prednisone doses, mg/day | | | 5 (3-5) | |  |  |  |  |  |  |
|  | NSAIDs | | | 194 (45) | |  |  |  |  |  |  |
|  | DMARDs | | | 373 (87) | |  |  |  |  |  |  |
|  | Methotrexate | | | 316 (73) | |  |  |  |  |  |  |
|  | Leflunomide | | | 94 (22) | |  |  |  |  |  |  |
|  | Hydroxychloroquine | | | 45 (18) | |  |  |  |  |  |  |
|  | Salazopyrin | | | 28 (7) | |  |  |  |  |  |  |
|  | Anti TNF therapy | | | 83 (19) | |  |  |  |  |  |  |
|  | Tocilizumab | | | 23 (5) | |  |  |  |  |  |  |
|  | Rituximab | | | 7 (2) | |  |  |  |  |  |  |
|  | Abatacept | | | 12 (3) | |  |  |  |  |  |  |
|  | JAK inhibitors | | | 20 (5) | |  |  |  |  |  |  |
|  | Baricitinib | | | 6 (1) | |  |  |  |  |  |  |
|  | Tofacitinib | | | 11 (3) | |  |  |  |  |  |  |
| Data represent mean ± SD or median (IQR) when data were not normally distributed. | | | | | | | | | | |  |
| CRP: C reactive protein; ACPA: Anti-citrullinated protein antibodies. | | | | | | | | | | |  |
| NSAID: Nonsteroidal anti-inflammatory drugs; DMARD: disease-modifying antirheumatic drug. | | | | | | | | | | |  |
| TNF: tumor necrosis factor; Obesity; ESR: erythrocyte sedimentation rate. | | | | | | | | | | |  |
| BMI: body mass index; DAS28: Disease Activity Score in 28 joints. | | | | | | | | | | |  |
| CDAI: Clinical Disease Activity Index; SDAI: Simple Disease Activity Index. | | | | | | | | | | |  |
