# Supplementary material for: Relationship between the complement system and serum lipid profile in patients with rheumatoid arthritis
Source: Front Immunol. 2024 Jul 12;15:1420292. doi: 10.3389/fimmu.2024.1420292 (PMC11272461; doi:10.3389/fimmu.2024.1420292)
Supplement: Supplementary file 2 [file Table_2.docx]

| **Supplementary Table 2. Functional test and individual elements values of the complement system and lipid profile in RA patients.** | | | |  |
| --- | --- | --- | --- | --- |
| Functional complement assays, % | | | |  |
| Classical pathway | | 96 ± 24 | |  |
| Alternative pathway | | 90 ± 26 | |  |
| Lectin pathway | | 50 (7-106) | |  |
| Individual complement components | | | |  |
| Classical pathway | |  | |  |
|  | C1q, mg/dl | 33 ± 8 | |  |
| Alternative pathway | |  | |  |
|  | Factor D, mg/dl | 0.17 ± 0.07 | |  |
|  | Properdin, mg/dl | 1.3 ± 0.4 | |  |
| Lectin pathway | |  | |  |
|  | Lectin, mg/dl | 0.08 (0.03-0.19) | |  |
| Classical and lectin pathways | | |  | |
|  | C1-inhibitor, mg/dl | 32 ± 7 | |  |
|  | C2, mg/dl | 7 (4-11) | |  |
|  | C4, mg/dl | 27 ± 10 | |  |
|  | C4b, mg/dl | 6 ± 3 | |  |
| Common pathway | |  | |  |
|  | C3, mg/dl | 141 ± 29 | |  |
|  | C3a, mg/dl | 34 ± 10 | |  |
|  | Factor I, mg/dl | 4 ± 1 | |  |
| Terminal pathway | |  | |  |
|  | C5, mg/dl | 3.9 ± 1.9 | |  |
|  | C5a, mg/dl | 1.0 (0.7-1.4) | |  |
|  | C9, mg/dl | 1.0 (0.6-1.3) | |  |
| Lipid profile | |  | |  |
| Cholesterol, mg/dl | | 206 ± 38 | |  |
| Triglycerides, mg/dl | | 147 ± 86 | |  |
| HDL, mg/dl | | 57 ± 15 | |  |
| LDL, mg/dl | | 120 ± 34 | |  |
| LDL:HDL ratio | | 2.27 ± 0.93 | |  |
| Non-HDL cholesterol, mg/dl | | 149 ± 39 | |  |
| Lipoprotein (a), mg/dl | | 34 (11-107) | |  |
| Apolipoprotein A1, mg/dl | | 174 ± 31 | |  |
| Apolipoprotein B, mg/dl | | 106 ± 26 | |  |
| Apo B:Apo A1 ratio | | 0.63 ± 0.19 | |  |
| Atherogenic index | | 3.88 ± 1.28 | |  |

HDL: High-density lipoprotein, LDL: Low-density lipoprotein

Data represent mean ± SD or median (IQR) when data were not normally distributed.
