# Supplementary material for: Relationship between the complement system and serum lipid profile in patients with rheumatoid arthritis
Source: Front Immunol. 2024 Jul 12;15:1420292. doi: 10.3389/fimmu.2024.1420292 (PMC11272461; doi:10.3389/fimmu.2024.1420292)
Supplement: Supplementary file 3 [file Table_3.docx]

| **Supplementary Table 3. Spearman's rho correlation coefficients between complement system routes and elements and lipoprotein profile molecules** | | | | | | | | | | | | | | | | | | | | | |  |
| --- | --- | --- | --- | --- | --- | --- | --- | --- | --- | --- | --- | --- | --- | --- | --- | --- | --- | --- | --- | --- | --- | --- |
|  |  | Cholesterol | Triglycerides | HDL | Non-HDL | | LDL | | LDL:HDL | ApoA1 | | ApoB | | ApoB:ApoA1 | | | Lpa | Atherogenic index | |  |  |  |
| Classical | Rho | -0.0234 | 0.0756 | 0.0147 | -0.0393 | -0.0626 | | -0.0454 | | | -0.0362 | | -0.0059 | | 0.0311 | 0.0372 | | | -0.0272 | |  |  |
| pathway | p | 0.6571 | 0.1515 | 0.7806 | 0.4562 | 0.2355 | | 0.39 | | | 0.4906 | | 0.911 | | 0.5552 | 0.4802 | | | 0.6056 | |  |  |
| C1q, mg/dl | Rho | 0.178 | **0.2285** | -0.1686 | **0.2285** | **0.1812** | | **0.2313** | | | 0.0403 | | **0.2109** | | 0.1309 | 0.0541 | | | **0.2418** | |  |  |
|  | p | 0.0508 | **0.0121** | 0.0646 | **0.0117** | **0.0476** | | **0.011** | | | 0.659 | | **0.0202** | | 0.1525 | 0.5555 | | | **0.0075** | |  |  |
| Alternative | Rho | 0.0095 | 0.0972 | -0.0147 | 0.0232 | 0.0002 | | 0.0283 | | | -0.0223 | | 0.0113 | | 0.0402 | 0.0955 | | | 0.0367 | |  |  |
| pathway | p | 0.8567 | 0.0652 | 0.7799 | 0.6594 | 0.9971 | | 0.5921 | | | 0.6719 | | 0.8308 | | 0.4447 | 0.0695 | | | 0.4861 | |  |  |
| Factor D, mg/dl | Rho | -0.058 | **0.1615** | **-0.1941** | 0.01 | -0.0432 | | 0.0648 | | | **-0.1917** | | 0.053 | | **0.1338** | 0.0313 | | | **0.1202** | |  |  |
|  | p | 0.2616 | **0.0017** | **0.0001** | 0.8473 | 0.4038 | | 0.2096 | | | **0.0002** | | 0.3047 | | **0.0093** | 0.5444 | | | **0.0196** | |  |  |
| Properdin | Rho | **0.2074** | **0.1254** | **-0.1249** | **0.253** | **0.2306** | | **0.2476** | | | -0.0892 | | **0.2952** | | **0.2874** | 0.0852 | | | **0.2333** | |  |  |
|  | p | **<0.001** | **0.015** | **0.0152** | **<0.001** | **<0.001** | | **<0.001** | | | 0.0829 | | **<0.001** | | **<0.001** | 0.0982 | | | **<0.001** | |  |  |
| Lectin pathway | Rho | -0.0293 | 0.0234 | -0.0872 | -0.015 | -0.0044 | | 0.033 | | | -0.1026 | | -0.0017 | | 0.0613 | 0.0496 | | | 0.0336 | |  |  |
|  | p | 0.5794 | 0.6588 | 0.0982 | 0.7758 | 0.9335 | | 0.5323 | | | 0.0508 | | 0.9737 | | 0.2447 | 0.3476 | | | 0.5246 | |  |  |
| Lectin, mg/dl | Rho | 0.0048 | -0.0298 | -0.0706 | 0.0166 | 0.0352 | | 0.055 | | | -0.0658 | | -0.0246 | | 0.0233 | 0.0438 | | | 0.0469 | |  |  |
|  | p | 0.9263 | 0.5652 | 0.1712 | 0.7473 | 0.4957 | | 0.2873 | | | 0.201 | | 0.6346 | | 0.6514 | 0.3957 | | | 0.3639 | |  |  |
| C1-inh | Rho | **0.1445** | **0.1192** | -0.0518 | **0.1561** | **0.1426** | | **0.145** | | | -0.0053 | | **0.1584** | | **0.1553** | **0.1082** | | | **0.1461** | |  |  |
|  | p | **0.0032** | **0.0153** | 0.2928 | **0.0014** | **0.0037** | | **0.0031** | | | 0.9142 | | **0.0012** | | **0.0015** | **0.0275** | | | **0.0029** | |  |  |
| C2, mg/dl | Rho | -0.0115 | -0.1076 | 0.0585 | -0.0227 | 0.013 | | -0.0106 | | | 0.0128 | | -0.0315 | | -0.015 | -0.0608 | | | -0.0479 | |  |  |
|  | p | 0.8426 | 0.0619 | 0.3101 | 0.6938 | 0.8216 | | 0.8542 | | | 0.8238 | | 0.5852 | | 0.7955 | 0.2915 | | | 0.4063 | |  |  |
| C4, mg/dl | Rho | 0.0679 | **0.1239** | -0.0081 | 0.0764 | 0.0483 | | 0.0562 | | | 0.016 | | **0.106** | | **0.102** | **0.0977** | | | 0.0682 | |  |  |
|  | p | 0.1686 | **0.0118** | 0.8691 | 0.121 | 0.3277 | | 0.2549 | | | 0.7463 | | **0.0315** | | **0.0385** | **0.0474** | | | 0.1663 | |  |  |
| C4b, mg/dl | Rho | 0.0482 | 0.0497 | 0.0261 | 0.0409 | 0.0253 | | 0.0127 | | | 0.037 | | 0.0786 | | 0.0551 | **0.1092** | | | 0.0064 | |  |  |
|  | p | 0.3508 | 0.3367 | 0.6134 | 0.429 | 0.6254 | | 0.8067 | | | 0.4721 | | 0.1278 | | 0.286 | **0.0339** | | | 0.9012 | |  |  |
| C3, mg/dl | Rho | **0.1672** | **0.3007** | -0.0909 | **0.2074** | **0.1151** | | **0.1621** | | | 0.0756 | | **0.2406** | | **0.1725** | **0.1288** | | | **0.215** | |  |  |
|  | p | **0.0006** | **<0.001** | 0.0651 | **<0.001** | **0.0195** | | **0.001** | | | 0.1246 | | **<0.001** | | **0.0004** | **0.0088** | | | **<0.001** | |  |  |
| C3a, mg/dl | Rho | -0.0323 | 0.144 | -0.097 | -0.0126 | -0.0252 | | 0.0269 | | | 0.0121 | | -0.0052 | | -0.0098 | 0.0448 | | | 0.0368 | |  |  |
|  | p | 0.7258 | 0.1181 | 0.2917 | 0.8915 | 0.7853 | | 0.7713 | | | 0.895 | | 0.9553 | | 0.9155 | 0.6272 | | | 0.6903 | |  |  |
| Factor I, mg/dl | Rho | 0.0728 | **0.148** | **-0.1654** | **0.1352** | 0.0983 | | **0.1776** | | | **-0.1346** | | **0.1896** | | **0.2391** | **0.1333** | | | **0.1938** | |  |  |
|  | p | 0.1584 | **0.004** | **0.0013** | **0.0086** | 0.057 | | **0.0005** | | | **0.0087** | | **0.0002** | | **<0.001** | **0.0095** | | | **0.0002** | |  |  |
| C5, mg/dl | Rho | 0.0344 | 0.0854 | -0.1 | 0.0624 | 0.0508 | | 0.0918 | | | -0.0829 | | **0.1054** | | **0.1537** | **0.1435** | | | **0.1029** | |  |  |
|  | p | 0.5071 | 0.0998 | 0.0532 | 0.2285 | 0.3282 | | 0.0765 | | | 0.1085 | | **0.0417** | | **0.0029** | **0.0054** | | | **0.0467** | |  |  |
| C5a, mg/dl | Rho | -0.0816 | **-0.2341** | 0.0042 | -0.0684 | 0.0548 | | 0.0367 | | | **-0.1232** | | 0.0154 | | 0.0886 | 0.0356 | | | -0.0368 | |  |  |
|  | p | 0.1138 | **<0.001** | 0.9351 | 0.1851 | 0.2893 | | 0.4778 | | | **0.0164** | | 0.7652 | | 0.0859 | 0.4903 | | | 0.4762 | |  |  |
| C9, mg/dl | Rho | -0.0912 | **-0.1028** | -0.0348 | -0.082 | -0.0307 | | 0.0013 | | | **-0.1288** | | -0.0323 | | 0.0772 | **0.1232** | | | -0.0254 | |  |  |
|  | p | 0.077 | **0.0463** | 0.5009 | 0.1118 | 0.5527 | | 0.98 | | | **0.0121** | | 0.5324 | | 0.1348 | **0.0165** | | | 0.6232 | |  |  |
| HDL: High-density lipoprotein, LDL: Low-density lipoprotein; ApoA1: Apolipoprotein A1, ApoB: Apolipoprotein B, Lpa: Lipoprotein (a), Atherogenic:  Atherogenic index. Significant p values are depicted in bold. | | | | | | | | | | | | | | | | | | | | | | |
