# Supplementary material for: Relationship between the complement system and serum lipid profile in patients with rheumatoid arthritis
Source: Front Immunol. 2024 Jul 12;15:1420292. doi: 10.3389/fimmu.2024.1420292 (PMC11272461; doi:10.3389/fimmu.2024.1420292)
Supplement: Supplementary file 4 [file Table_4.docx]

| **Supplementary Table 4. Multivariable analysis of the relation between complement system routes and elements and lipoprotein profile molecules.** | | | | | | | | | | | | | | | | | | | | | | | | | |
| --- | --- | --- | --- | --- | --- | --- | --- | --- | --- | --- | --- | --- | --- | --- | --- | --- | --- | --- | --- | --- | --- | --- | --- | --- | --- |
|  |  | | Cholesterol | | | Triglycerides | HDL | | Non-HDL | | LDL | | LDL:HDL | ApoA1 | | ApoB | | ApoB:ApoA1 | | | Lpa | Atherogenic index | | |  |
| Classical | | Std. β | |  | -0.0434 | | |  | |  |  |  | | |  | |  | |  |  | | |  |  |  |
| pathway | | p | |  | 0.43 | | |  | |  |  |  | | |  | |  | |  |  | | |  |  |  |
| C1q, mg/dl | | Std. β | | 0.1713 | 0.0205 | | | -0.1273 | | **0.2249** | **0.2550** | **0.3271** | | |  | | **0.2144** | | 0.1639 |  | | | **0.2303** |  |  |
|  | | p | | 0.079 | 0.82 | | | 0.17 | | **0.022** | **0.01** | **0.001** | | |  | | **0.029** | | 0.097 |  | | | **0.015** |  |  |
| Alternative | | Std. β | |  | -0.0382 | | |  | |  |  |  | | |  | |  | |  | 0.0639 | | |  |  |  |
| pathway | | p | |  | 0.51 | | |  | |  |  |  | | |  | |  | |  | 0.29 | | |  |  |  |
| Factor D, mg/dl | | Std. β | |  | 0.0402 | | | -0.1028 | |  |  |  | | | **-0.1577** | |  | | 0.0839 |  | | | 0.0471 |  |  |
|  | | p | |  | 0.46 | | | 0.059 | |  |  |  | | | **0.004** | |  | | 0.14 |  | | | 0.40 |  |  |
| Properdin | | Std. β | | **0.2251** | 0.0783 | | | -0.0844 | | **0.2572** | **0.2495** | **0.2547** | | | -0.0670 | | **0.3131** | | **0.0813** | 0.2199 | | | **0.2199** |  |  |
|  | | p | | **<0.001** | 0.12 | | | 0.096 | | **<0.001** | **<0.001** | **<0.001** | | | 0.17 | | **<0.001** | | **<0.001** | 0.13 | | | **<0.001** |  |  |
| Lectin | | Std. β | |  |  | | | -0.0096 | |  |  |  | | | -0.0213 | |  | |  |  | | |  |  |  |
| pathway | | p | |  |  | | | 0.85 | |  |  |  | | | 0.69 | |  | |  |  | | |  |  |  |
| Lectin, mg/dl | | Std. β | |  |  | | | 0.0050 | |  |  |  | | |  | |  | |  |  | | |  |  |  |
|  | | p | |  |  | | | 0.92 | |  |  |  | | |  | |  | |  |  | | |  |  |  |
| C1-inh | | Std. β | | **0.1297** | -0.0148 | | |  | | **0.1410** | **0.1651** | **0 .1053** | | |  | | **0.1675** | | **0.1351** | 0.0907 | | | 0.0633 |  |  |
|  | | p | | **0.013** | 0.77 | | |  | | **0.008** | **0.002** | **0.046** | | |  | | **0.002** | | **0.011** | 0.088 | | | 0.22 |  |  |
| C2, mg/dl | | Std. β | |  | -0.0379 | | |  | |  |  |  | | |  | |  | |  |  | | |  |  |  |
|  | | p | |  | 0.50 | | |  | |  |  |  | | |  | |  | |  |  | | |  |  |  |
| C4, mg/dl | | Std. β | | 0.0893 | -0.0141 | | |  | | 0.0086 |  |  | | |  | | 0.1100 | | 0.0767 | 0.0348 | | | 0.0220 |  |  |
|  | | p | | 0.11 | 0.79 | | |  | | 0.12 |  |  | | |  | | 0.050 | | 0.17 | 0.54 | | | 0.69 |  |  |
| C4b, mg/dl | | Std. β | |  |  | | |  | |  |  |  | | |  | | 0.0748 | |  | 0.1022 | | |  |  |  |
|  | | p | |  |  | | |  | |  |  |  | | |  | | 0.18 | |  | 0.068 | | |  |  |  |
| C3, mg/dl | | Std. β | | **0.2026** | **0.1308** | | | -0.0386 | | **0.2157** | **0.1786** | **0.1545** | | | 0.1072 | | **0.2750** | | **0.1436** | **0.1206** | | | **0.16037** |  |  |
|  | | p | | **0.001** | **0.022** | | | 0.51 | | **<0.001** | **0.003** | **0.010** | | | 0.068 | | **<0.001** | | **0.017** | **0.046** | | | **0.006** |  |  |
| C3a, mg/dl | | Std. β | |  | -0.0598 | | |  | |  |  |  | | |  | |  | |  |  | | |  |  |  |
|  | | p | |  | 0.56 | | |  | |  |  |  | | |  | |  | |  |  | | |  |  |  |
| Factor I, mg/dl | | Std. β | | 0.1166 | 0.0484 | | | -0.0853 | | **0.1503** | 0.1444 | **0.1734** | | | -0.0874 | | **0.2120** | | **0.2243** | 0.1127 | | | **0.1587** |  |  |
|  | | p | | 0.044 | 0.39 | | | 0.13 | | **0.010** | 0.014 | **0.003** | | | 0.13 | | **<0.001** | | **<0.001** | 0.056 | | | **0.006** |  |  |
| C5, mg/dl | | Std. β | |  | -0.0176 | | | -0.0051 | |  |  | 0.0540 | | | -0.0047 | | 0.0952 | | 0.0747 | **0.1743** | | | 0.0284 |  |  |
|  | | p | |  | 0.74 | | | 0.92 | |  |  | 0.33 | | | 0.93 | | 0.091 | | 0.19 | **0.002** | | | 0.61 |  |  |
| C5a, mg/dl | | Std. β | | 0.0248 | -0.0437 | | |  | | 0.0477 |  |  | | | -0.0726 | |  | | 0.0873 |  | | |  |  |  |
|  | | p | | 0.63 | 0.39 | | |  | | 0.36 |  |  | | | 0.16 | |  | | 0.10 |  | | |  |  |  |
| C9, mg/dl | | Std. β | | -0.1508 | **-0.1982** | | |  | | -0.1372 |  |  | | | **-0.1389** | |  | | 0.0504 | **0.2224** | | |  |  |  |
|  | | p | | 0.007 | **<0.001** | | |  | | 0.015 |  |  | | | **0.011** | |  | | 0.38 | **<0.001** | | |  |  |  |
| HDL: High-density lipoprotein, LDL: Low-density lipoprotein; ApoA1: Apolipoprotein A1, ApoB: Apolipoprotein B, Lpa: Lipoprotein (a), Atherogenic:  Atherogenic index. Significant p values are depicted in bold. Std. β: Standardized beta coefficients are adjusted for age,  sex, abdominal circumference, use of statins, anti-TNF therapies and tocilizumab, and DAS28-CRP. In this analysis complement system routes and elements and lipid profile are, respectively, the independent and dependent variable. Significant p values are depicted in bold. | | | | | | | | | | | | | | | | | | | | | | | | | |
